# A meta-analysis and systematic review of different cyclin-dependent kinase 4/6 inhibitors in breast cancer

**DOI:** 10.3389/fonc.2025.1472407

**Published:** 2025-03-04

**Authors:** Jialin Zhang, Xinyu Xu, Yeyue Zhou, Jingyang Su, Jue Wang

**Affiliations:** ^1^ Department of Oncology, Hangzhou TCM Hospital of Zhejiang Chinese Medical University (Hangzhou Hospital of Traditional Chinese Medicine), Hangzhou, China; ^2^ Department of General internal medicine, Tongde Hospital Affiliated to Zhejiang Chinese Medical University (Tongde Hospital of Zhejiang Province), Hangzhou, China

**Keywords:** breast cancer, CDK4/6 inhibitors, Abemaciclib, Palbociclib, Ribociclib, Dalpiciclib, adverse events

## Abstract

**Objective:**

The objective of this study was to assess the effectiveness and safety of CDK4/6 inhibitors in the treatment of hormone receptor-positive (HR+) breast cancer by using meta-analysis.

**Methods:**

To gather comprehensive and reliable data for our analysis, we systematically searched multiple databases for relevant studies. We utilized RevMan5.3 software to perform the meta-analysis.

**Results:**

Following a rigorous screening and evaluation process, we ultimately included a total of 13 studies in our analysis. Our findings showed that compared to endocrine therapy alone, the combination of CDK4/6 inhibitors with endocrine therapy significantly increased both PFS [HR 0.54 (95%CI: 0.50, 0.58), *P<0.00001*], OS [HR 0.77 (95%CI: 0.50, 0.58), *P<0.00001*] and ORR [RR 1.39 (95% CI: 1.21, 1.60), *P<0.00001*). However, it was also found that CDK4/6 inhibitors caused adverse drug reactions related to the blood system and digestive system (*P<0.0001*).

**Conclusions:**

Our meta-analysis demonstrates that the addition of CDK4/6 inhibitors to endocrine therapy can result in improved PFS and OS for HR+ breast cancer patients. Meanwhile, we recommend close monitoring and management of these potential side effects when utilizing these inhibitors in breast cancer treatment.

**Systematic Review Registration:**

https://www.crd.york.ac.uk/PROSPERO, identifier CRD42023490499.

## 1 Introduction

Breast cancer (BC) in women had surpassed lung cancer as the most common malignancy, with an estimated 2.3 million new cases (11.7%) in 2020 (1). Breast cancer can be divided into three subtypes: hormone receptor (HR)+, human epidermal growth factor receptor 2 (HER2) +, and triple negative subtypes. Among these, HR+ breast cancer is the most prevalent, accounting for approximately 70% of all cases (2, 3). A concerning aspect of breast cancer is its tendency to metastasize or spread to other parts of the body. This leads to a lower 5-year survival rate, which is less than 20%. It is, therefore, crucial to develop effective treatment strategies for metastatic breast cancer, particularly for HR+/HER2− subtypes (4). Endocrine therapy is considered the first-line treatment for HR+/HER2− metastatic breast cancer. However, one major challenge is the development of resistance to endocrine therapy, which significantly reduces its effectiveness.

In the mammary gland, BCL-2 is expressed in normal glandular epithelium, upregulated by estrogen, possibly as a result of direct transcriptional induction, and negatively regulated by p53-dependent mechanisms (5). BCL-2 is an anti-apoptotic gene, the expression of which can be used as a prognostic factor for breast cancer, and it is also thought to be associated with resistance to conventional cancer treatments (6, 7). In grade I to III breast cancer histology, the expression of BCL-2 showed a downward trend with significant differences (7). Through rank correlation analysis, Study has found a negative correlation between BCL-2 expression and chemotherapy sensitivity of breast cancer, suggesting that BCL-2 may make breast cancer cells resistant to chemotherapy drugs through its anti-apoptotic function (7). BCL-2 may be one of the efficacious prognostic factors that determine the efficacy of chemotherapy. In luminal A (HR+ and HER2-) and triple-negative subtypes, the expression of BCL-2 in tumor cells was significantly correlated with factors such as tumor size and tumor grade (8). In HER2 + breast cancer, BCL-2 expression is negatively correlated with c-erbB2 protein immunostaining and is a marker of poor prognosis (5). The expression of BCL-2 is mainly limited to ER-positive breast cancer cells, suggesting a good prognosis and that ER positivity is a necessary condition for endocrine therapy and may be more sensitive to endocrine therapy (5). Therefore, the detection of BCL-2 expression is conducive to providing a certain selection reference for the treatment of breast cancer patients with different pathological types. On the other hand, CDK4/6 inhibitor combined endocrine therapy has become one of the main therapeutic tools today, and it is also the main content of our research (9, 10).

One of the factors contributing to the uncontrolled proliferation of malignant tumor cells is the disruption of cell cycle regulation. Cyclin-Dependent Kinase 4/6 (CDK4/6) is a key regulator of the cell cycle, and CDK4 and CDK6 are a core part of cell cycle regulation, especially in the G1 phase when it forms a complex with cyclin D, leading to direct phosphorylation of the retinoblastoma gene and subsequent release of transcription factors (11). This mechanism promotes the transition of the cell cycle from the G1 phase to the S phase, thereby inhibiting the cell’s DNA replication and cell division (12). Estrogen activates signaling pathways of ER, especially in estrogen receptor (ER)+ breast cancer, resulting in the upregulation of the expression of cyclin D and CDK4/6 (13, 14). In light of this mechanism, CDK4/6 inhibitors have emerged as potential therapeutic options for ER+ breast cancer. CDK4/6 inhibitors induce the increase of abnormal proteins on the surface of tumor cells, so that they are recognized and cleared by the immune system, and regulate the tumor microenvironment by affecting the activity of tumor secreted cytokines and T cells, and further inhibit the growth and spread of tumors. Meanwhile, CDK4/6-inhibited cells can also overgrow during G0/G1, leading to p53-dependent cell cycle exit (15). By restoring the cell cycle and blocking cell proliferation, these inhibitors can effectively inhibit the progression of ER+ breast cancer. Both preclinical research and clinical trials have provided evidence of the effectiveness of CDK4/6 inhibitors (Palbociclib, Ribociclib, and Abemaciclib) in treating HR+ BC. As a result, regulatory authorities including the U.S. FDA and other global pharmaceutical agencies have granted licenses for the use of CDK4/6 inhibitors in combination with endocrine therapy or as standalone treatments (Abemaciclib) for the initial management of HR+/HER2– breast cancer patients (16). To further evaluate the effectiveness and potential adverse events of different types of CDK4/6 inhibitors in the treatment of malignant tumors, we performed a meta-analysis by aggregating data from multiple randomized controlled trials. The aim is to compare the efficacy and adverse events of different CDK4/6 inhibitors in breast cancer, and weigh the advantages and disadvantages, in order to provide more clinical drug reference for readers.

## 2 Methods

### 2.1 Search strategy

We followed the guidelines set by PRISMA to conduct our research. To ensure a comprehensive search, we systematically looked for relevant studies in multiple databases including PubMed, Cochrane, and Embase. Our search covered data up until November 30, 2023, to include the most recent information available. Additionally, registration on PROSPERO (No. CRD42023490499) was completed. To optimize the search process, we used a combination of Medical Subject Headings (MeSH) terms and Free terms. This helped us to capture a wide range of articles that fulfilled our research criteria. The detailed search strategy, including the specific terms we used, can be found in **Supplementary Appendix 1**. The relevant search terms for the participants and intervention factors are as follows:

1. Patients: the MeSH term is “Breast Neoplasms”, the free terms are ((Breast Neoplasm) OR (Breast Tumor) OR (Breast Cancer) OR (Breast Carcinoma) OR (Mammary Cancer) OR (Mammary Carcinoma) OR (Mammary Neoplasm) OR (Mammary Tumor).
2. Intervention: Drugs include CDK4/6 inhibitors, the free terms are (Abemaciclib) OR (Palbociclib) OR (Dalpiciclib) OR (Trilaciclib) OR (Ribociclib).

### 2.2 Study designs

Our article focused exclusively on Randomized Controlled Trials (RCTs) as the study design for evaluating the efficacy of different CDK4/6 inhibitors in treating advanced breast cancer. Other types of study designs, such as cohort analyses, cross-sectional studies, case-reports, and single-arm trials, were not included in our review. Furthermore, we excluded animal studies, incomplete or replicated clinical studies, and studies with small sample sizes. Additionally, studies for which the full text was not available were also excluded from our analysis.

#### 2.2.1 Participants/patients

For this study, we enrolled participants who had been diagnosed with breast cancer through definitive cytologic or histologic methods. All the patients in studies were HR+ breast cancer. We did not set an age cutoff, as our goal was to include as broad a population as possible.

#### 2.2.2 Intervention and comparison

In our chosen studies, the intervention group received treatment with CDK4/6 inhibitors in combination with endocrine therapy. While the control group received a placebo in addition to endocrine therapy. To ensure consistency and comparability of the treatment conditions, it was crucial for the control group to receive the same endocrine therapy as the intervention group.

#### 2.2.3 Outcomes

Primary outcome: The main measure we used to evaluate the effectiveness of the intervention was progression-free survival (PFS). Secondary outcomes: In addition to PFS, we also analyzed overall survival (OS) and objective response rate (ORR) as secondary outcomes. Safety indicator: To assess the safety profile of the intervention, we considered adverse events (AE) as an important indicator.

### 2.3 Data extraction

The process of data extraction involved two authors (referred to as JS and YZ) screening all studies independently, including titles, keywords, and abstracts, based on the inclusion criteria. Any uncertainties or potential questions were settled by a third author (referred to as JW). After the initial screening, the two authors continued to read the full text of the selected studies and conducted a secondary screening. Finally, they cross-checked the included studies to ensure accuracy. The extracted information from the articles included several key details. These details included the name of the first author, the publication year, the specific study design that was employed, the number of participants in each study, the treatment used in the intervention group and control group (including details such as median PFS and OS), as well as outcomes related to different pathological types and any major adverse events that were reported.

### 2.4 Assessment of risk of bias

To assess the risk of bias in the included studies, two evaluators (JS and YZ) independently used the Cochrane Risk of Bias tool. This tool allowed them to evaluate several potential sources of bias. These sources included bias during the randomization process, bias that may have been caused by inadequate allocation concealment, bias arising from the use of open and non-blind parallel methods, bias resulting from participants being lost to follow-up or missing data, and bias in the selection of primary or secondary outcomes. By assessing these various sources of bias, the evaluators were able to determine the overall risk of bias in each included study. All the studies included in our article were large, high-quality randomized controlled double-blind trials without high risk, as shown in **Table 1**.

**Table 1:** Risk-of-Bias table of included studies.

NO.	Study	Random sequence generation	Allocation concealment	Blinding of participants and personnel	Blinding of outcome assessment	Data integrity	Selective reporting	Other bias
1	Johnston 2019 (17)	Low risk	Low risk	Low risk	Low risk	Low risk	Low risk	Low risk
2	Sledge 2020 (18)	Low risk	Low risk	Low risk	Low risk	Low risk	Low risk	Low risk
3	Zhang 2020 (19)	Low risk	Low risk	Low risk	Low risk	Low risk	Low risk	Low risk
4	Zhang 2020 (19)	Low risk	Low risk	Low risk	Low risk	Low risk	Low risk	Low risk
5	Xu 2021 (20)	Low risk	Unclear risk	Low risk	Low risk	Low risk	Low risk	Low risk
6	Zhang 2023 (21)	Low risk	Low risk	Low risk	Low risk	Low risk	Low risk	Low risk
7	Hortobagyi 2022 (22)	Low risk	Low risk	Low risk	Low risk	Low risk	Low risk	Low risk
8	Lu 2022 (23)	Low risk	Low risk	Low risk	Low risk	Low risk	Low risk	Low risk
9	Neven 2023 (24)	Low risk	Low risk	Low risk	Low risk	Low risk	Low risk	Low risk
10	Albanell 2022 (25)	Low risk	Low risk	Low risk	Low risk	Low risk	Low risk	Low risk
11	Finn 2016 (26)	Low risk	Low risk	Low risk	Low risk	Low risk	Low risk	Low risk
12	Finn 2020 (27)	Low risk	Unclear risk	Low risk	Low risk	Low risk	Low risk	Low risk
13	Turner 2018 (28)	Low risk	Low risk	Low risk	Low risk	Low risk	Low risk	Low risk
14	Xu 2022 (29)	Low risk	Unclear risk	Low risk	Low risk	Low risk	Low risk	Low risk

### 2.5 Statistical method and analysis

The data obtained from the research studies were carefully analyzed using the highly regarded RevMan 5.3 software. The primary outcomes of PFS and OS were accurately measured and expressed as Hazard Ratios (HR) along with their corresponding 95% Confidence Intervals (CI). If HR < 1 and *P*-value is significant, it indicates that the control group has better efficacy. If HR = 1, there is no significant difference in efficacy between the two regimens. If HR > 1 and *P*-value is significant, the intervention group is less effective. The secondary outcomes, such as ORR and AEs, were presented as Risk Ratios (RR) along with their 95% CIs. To assess the presence of heterogeneity among the studies, two statistical methods were employed. To assess the heterogeneity of the results, chi-square (χ2) test (Cochran’s Q) and inconsistency index (*I^2^*) were used. The heterogeneity was high when the χ2 *P* value was less than 0.1 or the *I^2^* was more than 50%. For outcomes with significant heterogeneity (χ2 *P* value less than 0.1 or *I^2^* greater than 50%), random effects models were employed to calculate the total HR or RR. However, if there was no significant heterogeneity, fixed effects models were applied. To further explore the sources of high heterogeneity, a sensitivity analysis was conducted. This involved systematically excluding individual studies from the analysis to assess their impact on the overall pooling of data. By doing so, we could identify whether any single study was disproportionately influencing the overall results. Alternatively, subgroup analysis was performed to investigate the potential causes of high heterogeneity. By analyzing each subgroup separately, we hoped to uncover any underlying factors that might explain the observed heterogeneity.

## 3 Results

### 3.1 Search process

**Figure 1:**
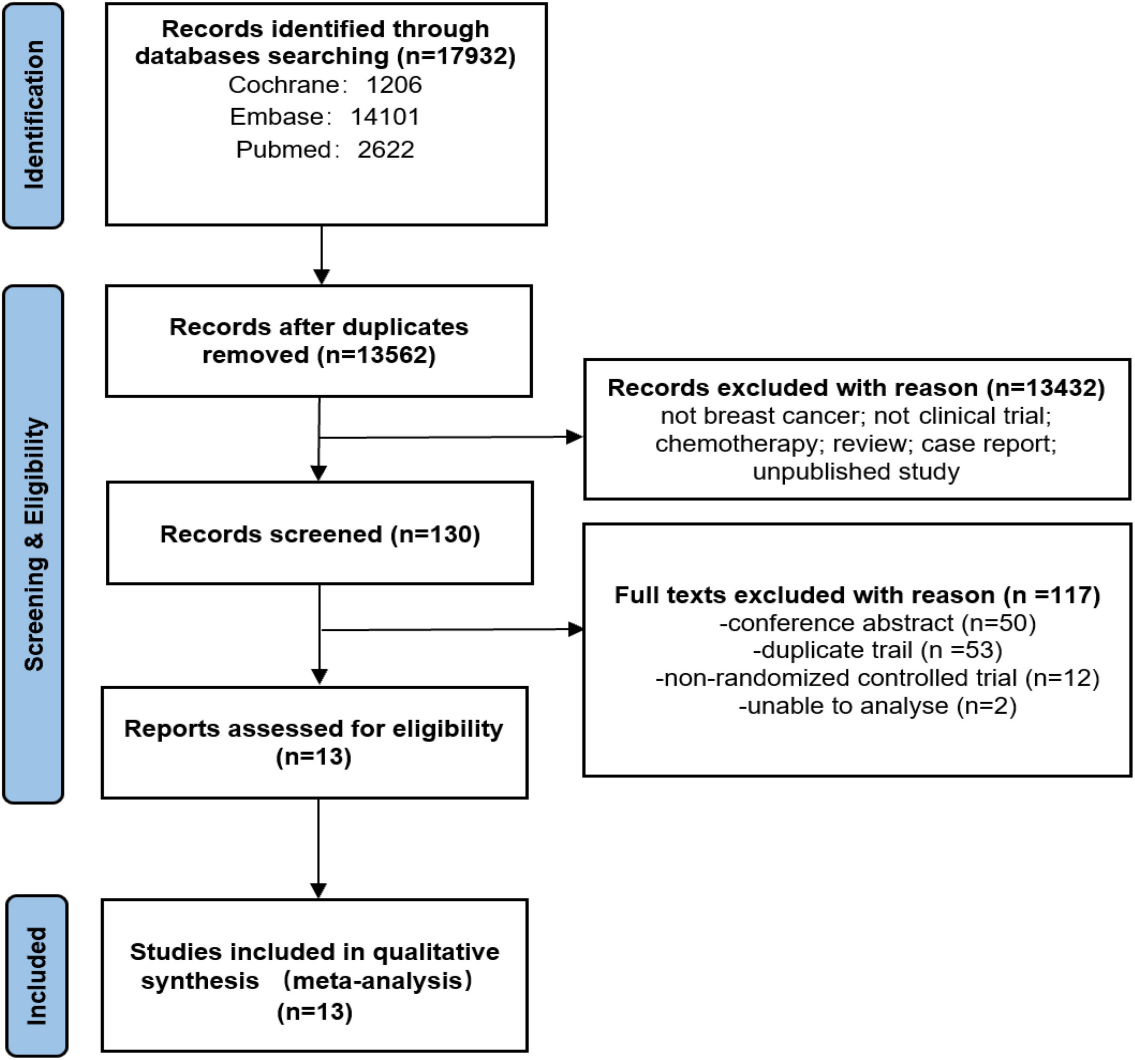
PRISMA flow diagram of search.

The initial search process involved searching through three databases. From this search, a total of 17932 articles were deemed relevant. However, after removing duplicate articles (4370), and excluding 13432 articles based on title, key words, abstract, only 130 potential studies remained for further analysis. Finally, only 13 studies were included for meta-analysis. The details of this process are illustrated in **Figure 1**.

### 3.2 Quality assessment

Whether it was phase II or phase III clinical trial, all the 13 included studies were randomized controlled double-blind trials, and all of them were high-quality clinical studies with reliable and valid data.

### 3.3 Study characteristics

**Table 2** presents the key patient characteristics of the studies included in the analysis. All eligible patients were over the age of 18.

**Table 2:** Overview of studies’ characteristics of different CDK4/6.

	Study	Classification (intervention group)	Classification (control group)
1	Johnston 2019 (17)	Patients:328 people; Drugs: Abemaciclib+NSAI Outcomes: Median PFS was 28.18 months; 9CR; 154PR	Patients:165 people; Drugs: Placebo+NSAI Outcomes: Median PFS was 14.76 months; 1CR; 60PR
2	Sledge 2020 (18)	Patients:446 people; Drugs: Abemaciclib+Fulvestrant Outcomes: Median PFS was 16.4 months; OS was 46.7 months; 14CR; 143PR	Patients:223 people; Drugs: Placebo+Fulvestrant Outcomes: Median PFS was 9.3 months; OS was 37.3 months; 1CR; 35PR
3	Zhang 2020 (19)	Patients: 207 people; Drugs: Abemaciclib+NSAI Outcomes: Median PFS was NR; 2CR; 114PR	Patients: 99 people; Drugs: Placebo+NSAI Outcomes: Median PFS was 14.7 months; 0CR; 63PR
4	Zhang 2020 (19)	Patients:104 people; Drugs: Abemaciclib+Fulvestrant Outcomes: Median PFS was 11.5 months; 0CR; 40PR	Patients: 53people; Drugs: Placebo+Fulvestrant Outcomes: Median PFS was 5.6 months; 1CR; 3PR
5	Xu 2021 (20)	Patients: 241 people; Drugs: Dalpiciclib+Fulvestrant Outcomes: Median PFS was 15.7 months; OS was NR; 2CR; 63PR	Patients: 120 people; Drugs: Placebo+Fulvestrant Outcomes: Median PFS was 7.2 months; OS was 14.2 months; 0CR; 24PR
6	Zhang 2023 (21)	Patients: 303 people; Drugs: Dalpiciclib+Letrozole or Anastrozole Outcomes: Median PFS was 30.6 months; 2CR; 172PR	Patients: 153 people; Drugs: Placebo+Letrozole or Anastrozole Outcomes: Median PFS was 18.2 months; 0CR; 73PR
7	Hortobagyi 2022 (22)	Patients: 334 people; Drugs: Ribociclib+Letrozole Outcomes: Median PFS was 25.3 months; OS was 63.9 months; 13CR; 129PR	Patients: 334 people; Drugs: Placebo+Letrozole Outcomes: Median PFS was 16.0 months; OS was 51.4 months; 8CR; 88PR
8	Lu 2022 (23)	Patients: 335 people; Drugs: Ribociclib+Endocrinotherapy Outcomes: Median PFS was 23.8 months; OS was 58.7 months; 8CR; 129PR	Patients: 337 people; Drugs: Placebo+Endocrinotherapy Outcomes: Median PFS was 13.0 months; OS was 48.0 months; 7CR; 93PR
9	Neven 2023 (24)	Patients: 484 people; Drugs: Ribociclib+Fulvestrant Outcomes: Median PFS was 20.5 months; OS was 67.6 months; 8CR; 149PR	Patients: 242 people; Drugs: Placebo+Fulvestrant Outcomes: Median PFS was 12.8 months; OS was 51.8 months; 0CR; 52PR
10	Albanell 2022 (25)	Patients: 94 people; Drugs: Palbociclib+Fulvestrant Outcomes: Median PFS was 31.8 months; 1CR; 42PR	Patients: 95 people; Drugs: Placebo+Fulvestrant Outcomes: Median PFS was 22.0 months; 4CR; 26PR
11	Finn 2016 (26)	Patients: 444 people; Drugs: Palbociclib+Letrozole Outcomes: Median PFS was 31.8 months; 187 (CR+PR)	Patients: 222 people; Drugs: Placebo+Letrozole Outcomes: Median PFS was 14.5 months; 77 (CR+PR)
12	Finn 2020 (27)	Patients: 84 people; Drugs: Palbociclib+Letrozole Outcomes: Median PFS was 20.2 months; OS was 37.5 months; 1CR; 35PR	Patients: 81 people; Drugs: Placebo+Letrozole Outcomes: Median PFS was 10.2 months; OS was 34.5 months; 1CR; 26PR
13	Turner 2018 (28)	Patients: 347 people; Drugs: Palbociclib+Fulvestrant Outcomes: Median PFS was 9.5 months; OS was 34.9 months; 0CR; 66PR	Patients: 174 people; Drugs: Placebo+Fulvestrant Outcomes: Median PFS was 4.6 months; OS was 28 months; 4CR; 11PR
14	Xu 2022 (29)	Patients: 169 people; Drugs: Palbociclib+Letrozole Outcomes: Median PFS was 21.5 months; 2CR; 61PR	Patients: 171 people; Drugs: Placebo+Letrozole Outcomes: Median PFS was 13.9 months; 1CR; 53PR

PFS, progression-free survival; OS, overall survival; CR, complete response; PR, partial response.

### 3.4 Outcomes

#### 3.4.1 Progression-free survival

**Figure 2:**
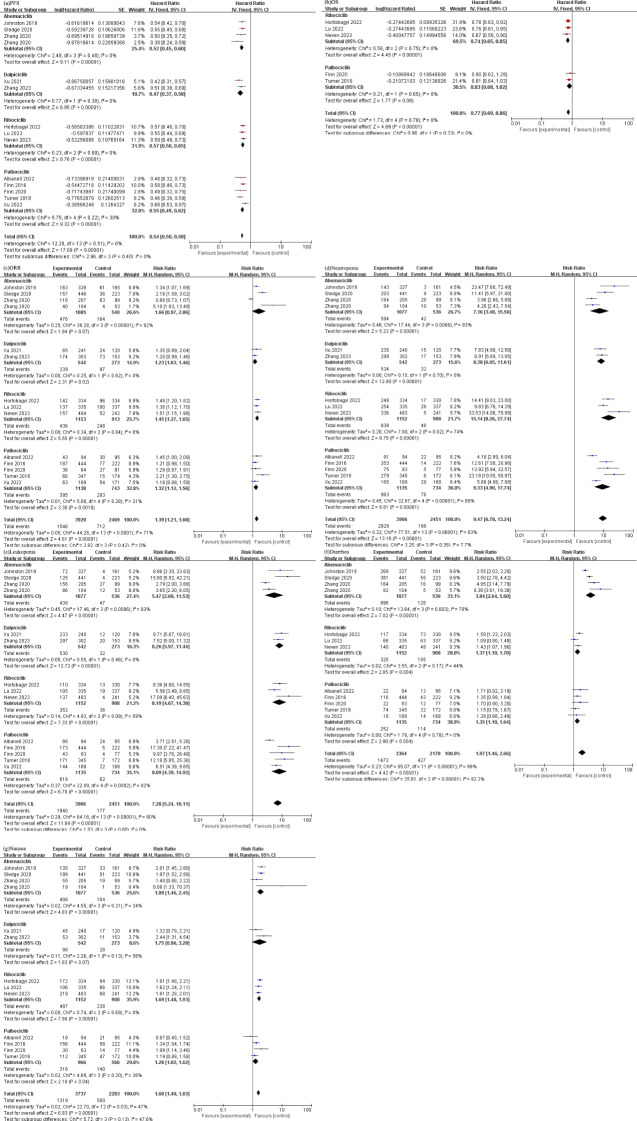
**(a)** Forest plot of the PFS of CDK4/6 inhibitors in patients with BC. **(b)** Forest plot of the OS of CDK4/6 inhibitors in patients with BC. **(c)** Forest plot of the ORR of CDK4/6 inhibitors in patients with BC. **(d)** Forest plot of the neutropenia of CDK4/6 inhibitors. **(e)** Forest plot of the leukopenia of CDK4/6 inhibitors. **(f)** Forest plot of the diarrhea of CDK4/6 inhibitors. **(g)** Forest plot of the nausea of CDK4/6 inhibitors.

All RCTs included in the study reported the progression-free survival (PFS) of the patients. The combined analysis of these studies revealed a statistically significant prolongation of PFS in patients treated with CDK4/6 [HR 0.54 (95%CI: 0.50, 0.58), *P<0.00001*], and subgroup analysis showed that there was no heterogeneity in each drug group, as depicted in **Figure 2A**.

#### 3.4.2 Overall survival

The pooled results from various studies also demonstrated a significant improvement and low heterogeneity in overall survival (OS) among individuals who received CDK4/6 [HR 0.77 (95%CI: 0.69, 0.86), *P<0.00001*], as presented in **Figure 2B**.

#### 3.4.3 Objective response rate

The analysis of ORR indicated a significant increase in the likelihood of achieving an objective response in patients treated with CDK4/6 [RR 1.39 (95%CI: 1.21, 1.60), *P<0.00001*], as graphically presented in **Figure 2C**.

#### 3.4.4 Adverse events

We extracted common adverse events of studies and divided these into the hematologic system and the gastrointestinal system, as demonstrated in **Figures 2D-G**). We extracted and analyzed the diseases of each system from randomized controlled trials as follow: (1) Hematologic system: (Neutropenia: [RR 9.47, (95% CI: 6.78, 13.24), *P<0.00001*]; Leukopenia: [RR 7.28, (95% CI: 5.24, 10.11), *P<0.00001*]). (2) Gastrointestinal system: (Diarrhea: [RR 1.97, (95% CI: 1.46, 2.66), *P<0.00001*]; Nausea: [RR 1.60, (95% CI: 1.40, 1.83), *P<0.00001*).

## 4 Discussion

In our study, we focused on analyzing a group of patients diagnosed with HR+/HER2- breast cancer and their response to different CDK4/6 inhibitors. In this meta-analysis, we compared different CDK4/6 inhibitor’s primary efficacy when used in combination with endocrine therapy, including Abemaciclib, Dalpiciclib, Ribociclib, and Palbociclib (17–29). To assess the effectiveness of these CDK4/6 inhibitors, we evaluated three key parameters: progression-free survival (PFS), overall survival (OS), and objective response rate (ORR). By comparing these outcomes between the different CDK4/6 inhibitors and endocrine therapy alone, we were able to draw some significant conclusions. Our results clearly demonstrated that CDK4/6 inhibitors plus endocrine therapy had a substantial positive impact on both PFS and OS in patients with HR+/HER2- breast cancer and had no heterogeneity compared with endocrine therapy alone. When examining the individual performance of each CDK4/6 inhibitor, we found that Dalpiciclib showed the most promising results in terms of PFS. Compared with the control group, the risk of death in patients treated with Dalpiciclib was 47% [HR 0.47 (95%CI: 0.37, 0.58), P<0.00001]. Abemaciclib, Palbociclib, and Ribociclib also demonstrated favorable outcomes, with risk of 52% [HR 0.52 (95%CI: 0.45, 0.60), P<0.00001], 55% [HR 0.55 (95%CI: 0.49, 0.62), *P<0.00001*], and 57% [HR 0.57 (95%CI: 0.50, 0.65), *P<0.00001*] respectively. Moreover, the similarities in efficacy among the different CDK4/6 inhibitors suggest that they can all be effective treatment options in this patient population. Abemaciclib, Palbociclib, and Ribociclib are three well-established compounds that have undergone extensive clinical trials. These compounds have shown high selectivity for CDK4 and CDK6 (30). Abemaciclib has been found to inhibit CDK4/6 at low nanomolar concentrations. It has been demonstrated to reduce the phosphorylation of RB1, a tumor suppressor protein, leading to the induction of G1 phase arrest in colorectal cancer (31). Additionally, Abemaciclib has shown the ability to reduce the activity of CDK9, another cyclin-dependent kinase (32). Palbociclib, on the other hand, primarily inhibits CDK4 and CDK6 at low nanomolar concentrations. It has limited inhibition against other CDKs or tyrosine kinases. In studies using breast cancer models, Palbociclib has shown synergistic inhibitory effects when combined with other therapies such as trastuzumab or endocrine Therapy. In the presence of CDK4/6 inhibition alone, sustained cyclin E2 expression continues to allow low levels of S-phase entry and a synergistic effect with endocrine therapy can be observed by inhibition of residual cyclin (30, 33). Furthermore, Palbociclib has been found to arrest the growth of breast cancer cell lines that have developed resistance to endocrine therapy, as these cells still rely on the activation of CDK4/6 (34). Ribociclib, like Abemaciclib and Palbociclib, inhibits CDK4 and CDK6 at nanomolar concentrations. It has been shown to inhibit the growth of neuroblastoma and liposarcoma cell lines, leading to G1 phase arrest. Ribociclib has also been found to reduce the phosphorylation of RB1 at specific sites, Ser780 and Ser807/811 (35, 36). These findings have been validated in xenograft models of neuroblastoma and liposarcoma, where Ribociclib significantly reduced tumor burden.

Previous meta-study results show CDK4/6 improves OS in breast cancer patients (37), but OS data remain incomplete, only some studies of Ribociclib and Palbociclib have reported the results of OS, with risk reductions of 26% [HR 0.74 (95%CI: 0.65, 0.85), *P<0.00001*] and 17% [HR 0.83 (95%CI: 0.68, 1.02), *P<0.00001*], respectively. One of the MONAECH-2 (18) study of Abemaciclib and the DAWNA-1 (20) study of Dalpiciclib also reported the OS, which was 46.7vs 37.3 months [HR 0.757 (95%CI: 0.606, 0.945), *P=0.01*] and NR vs 14.2 months [HR 0.47 (95%CI: 0.32, 0.69), *P<0.0001*] in the intervention and control groups, respectively. However, meta-analysis could not be performed because of only one study result of each drug. Furthermore, the results of ORR showed that Dalpiciclib, Ribociclib and Palbociclib combined with endocrine therapy significantly improved ORR compared with endocrine therapy alone without heterogeneity. However, in the Abemaciclib group, the results showed heterogeneity due to the large difference in ORR results among the studies. This discrepancy may be attributed to the fact that Abemaciclib combined with fulvestrant (17, 19) demonstrated better efficacy than using non-steroidal aromatase inhibitor (NSAI) (18, 19). Nevertheless, it is also possible that some patients developed drug resistance or transitioned from being sensitive to endocrine treatment to becoming resistant, which consequently led to poor efficacy. A network meta-analysis (38) confirmed that the addition of CDK4/6 inhibitors to fulvestrant significantly increased the clinically important endpoint of OS, regardless of whether the patients were endocrine-sensitive or endocrine-resistant. This implies that CDK4/6 inhibitors, when combined with fulvestrant, had a meaningful impact in improving overall survival rates. Especially, in the case of endocrine resistance, the combination of CDK4/6 inhibitors with fulvestrant may offer the best treatment option for patients with visceral diseases.

CDK4/6 combined with endocrine therapy is also better than chemotherapy alone. In the KCSG-BR15-10 study (39), Palbociclib with endocrine therapy (exemestane) and gonadotropin-releasing hormone agonist was found to be more effective than chemotherapy alone. The study showed that patients who received Palbociclib plus s exemestane with gonadotropin-releasing hormone agonist had a significantly longer PFS compared to those who received capecitabine as chemotherapy. The median PFS for the Palbociclib group was 20.1 months, while it was only 14.4 months for the capecitabine group, [HR 0.659 (95%CI: 0.437, 0.994), *P<0.05*]. Similarly, in the CORALLEEN trial (40), the combination of another CDK4/6 inhibitor (Ribociclib) with letrozole was found to be more effective than chemotherapy in downstaging high-risk luminal B breast cancer. In summary, CDK4/6 combined endocrine therapy is still the first choice for first-line endocrine therapy for endocrine-sensitive HR+ advanced breast cancer (41). In addition to the previously mentioned CDK4/6 inhibitors, Dinaciclib and Trilaciclib have also shown promising potential in treating cancer. Recent data from a phase II study (42) revealed that Trilaciclib demonstrated impressive antitumor effects, achieving an overall survival rate of 20.1 months in one cohort and 17.8 months in another cohort that received Trilaciclib treatment. In comparison, the chemotherapy group, which was treated with gemcitabine and carboplatin alone, only achieved a median overall survival of 12.6 months. However, Dinaciclib’s efficacy as a monotherapy was not found to be superior to capecitabine (43). While Dinaciclib did show some antitumor activity, it did not outperform capecitabine in this study. It is worth noting that neither Dinaciclib nor Trilaciclib were not included in the meta-analysis because there was only one study data for each.

The adverse reactions of CDK4/6 inhibitors primarily affect the hematological system, including Neutropenia and Leukopenia, followed by the digestive system. These adverse events were observed to be significantly higher in the CDK4/6 inhibitor group compared to the control group, indicating a strong correlation with bone marrow suppression caused by the CDK inhibitor’s ability to arrest cell-cycle reversibly. In terms of Neutropenia, the meta-analysis results revealed that patients who received Ribociclib had the highest incidence rate (RR=15.14), followed by Palbociclib (RR=9.33), Dalpiciclib (RR=8.38), and Abemaciclib (RR=7.36). For Leukopenia, Dalpiciclib (RR=8.26), Ribociclib (RR=8.19), and Palbociclib (RR=8.09) had similar incidence rates, while Abemaciclib (RR=5.47) had the lowest incidence. However, it is important to note that a previous meta-analysis showed a higher frequency of adverse events, serious adverse events, and deaths due to adverse events in patients who received Abemaciclib compared to Ribociclib and Palbociclib (44). This is attributed to the poor gastrointestinal tolerance of Abemaciclib, leading to gastrointestinal toxicity (45, 46). Among the CDK4/6 inhibitors, Abemaciclib stood out as having the highest incidence of diarrhea. In fact, the majority (42.8%) of patients who received Abemaciclib reported clinically significant diarrhea (grade ≥2) (45). This finding is corroborated by the significantly higher incidence of diarrhea with Abemaciclib (RR=3.84) compared to other CDK4/6 inhibitors, as observed in our meta-analysis results. Concurrent nausea was significantly higher in the CDK4/6 inhibitor group without heterogeneity. It is crucial for clinicians and patients to have a thorough understanding of drug reactions before starting clinical use in order to prevent the occurrence of serious drug-related adverse events. Grade ≥3 neutropenia, typically occurs within the first two cycles of treatment with CDK4/6 inhibitors, and it can be resolved by adjusting the dosage (45). However, when neutropenia is accompanied by fever, it is referred to as febrile neutropenia and requires intervention with granulocyte colony-stimulating factor (G-CSF) for patients (47). The use of G-CSF not only helps to improve the patients’ current quality of life by alleviating the symptoms of neutropenia but also helps to better prepare them for the subsequent course of treatment. Diarrhea is the most commonly observed adverse event associated with Abemaciclib treatment. Usually, it occurs within the first 7 days of starting the medication, but it can be managed effectively by using antidiarrheal agents and adjusting the dosage within a period of 2 weeks (45). Patients should be informed about the possibility of early-onset diarrhea, and they should promptly start antidiarrheal agents at the first signs of loose stools. If the symptoms do not improve within 24 hours of initiating antidiarrheal therapy, a dosage adjustment should be considered after discussing with a healthcare provider. A disproportionality analysis suggested that the identification of signals of disproportionality may help increase awareness of toxicities for Abemaciclib, whereas the time-to-onset, serious and nonserious reporting, and clinical priority analyses provided some supportive evidence for clinicians in their management of adverse events (48). Additionally, for patients experiencing nausea, stomach protection measures and antiemetic drugs can be used as a preventive measure before taking the medication to prevent the aggravation of nausea symptoms.

We are the first study to discuss the efficacy and adverse effects of different CDK4/6 inhibitors in a subgroup analysis with objective, reliable and statistically significant results. But there are some limitations to our study. First of all, our study involved a large number of drugs but included fewer clinical trials for each drug, and the sample size was small, which limited our evaluation of OS results. Secondly, the differences in the population involved in different drugs may cause some bias. For example, the two studies related to Dalpiciclib only involved Chinese people, and further research is needed. Finally, the efficacy and safety differences between the two CDK4/6 inhibitors were not directly compared. Both BCL-2 and p53 mRNA and protein levels are reduced in breast cancer brain metastases, suggesting that monitoring the expression of BCL-2 and p53 could serve as a prognostic tool (49). Furthermore, the antisense oligonucleotide targeting BCL-2 effectively downregulates BCL-2 expression in breast cancer cells, reducing its inhibitory effect on chemotherapy-induced apoptosis and enhancing therapeutic efficacy. This approach offers a promising new avenue for treating breast cancer. Looking ahead, combining dual-targeted therapies involving CDK4/6 and the BCL-2 pathway may provide an effective strategy for treating HR+ breast cancer. Previous studies have shown that inhibition of BCL-2 and CDK4/6 combined with endocrine therapy can inhibit proliferation and induce apoptosis of cancer cells, including phenotype senescent cells, thereby enhancing reactivity *in vivo* (10). The downregulation of ER after treatment with BCL-2 inhibitors is consistent with the resulting G1 block, which may lessen resistance to CDK4/6 inhibitors (50). These findings suggest the potential of BCL-2 and CDK4/6 dual-targeted drug combinations as early first-line therapy for patients with endocrine-sensitive tumors, aimed at delaying adaptive resistance, but more data are needed to support this.

## 5 Conclusion and future directions

CDK4/6 inhibitors, combined with endocrine therapy, have emerged as the primary treatment option for patients with hormone receptor-positive (HR+) and HER2-negative breast cancer. This treatment approach has shown significant success in improving patient outcomes. Currently, there are several CDK4/6 inhibitors available in the market, offering a range of options for physicians to choose from. Some of the CDK4/6 inhibitors that have gained FDA approval and are widely used include Abemaciclib, Palbociclib, and Ribociclib. These inhibitors have demonstrated their efficacy in extending PFS and OS in HR+/HER2- breast cancer patients. However, it is crucial to note that different inhibitors have distinct side effects profiles. For example, Palbociclib, Ribociclib, and Dalpiciclib are known to have a higher likelihood of bone marrow suppression, leading to neutropenia and leukopenia. This highlights the importance of monitoring patients’ blood counts regularly during treatment with these inhibitors to prevent complications and adjust dosage if necessary. On the other hand, Abemaciclib is primarily associated with gastrointestinal toxicity, particularly diarrhea. Recognizing and appropriately managing these adverse reactions early on is essential not only for improving the patients’ quality of life but also for optimizing their overall prognosis and follow-up care. In conclusion, the combination of CDK4/6 inhibitors with endocrine therapy has revolutionized the treatment landscape for HR+/HER2- breast cancer patients. While each CDK4/6 inhibitor offers its unique advantages, it is crucial to consider their distinct side effects and take appropriate measures to manage them effectively. By doing so, healthcare professionals can maximize the benefits of these inhibitors, leading to improved outcomes and overall patient satisfaction.

## Data Availability

The original contributions presented in the study are included in the article/**Supplementary Material**. Further inquiries can be directed to the corresponding author.

## References

[1] Sung H Ferlay J Siegel RL Laversanne M Soerjomataram I Jemal A . Global cancer statistics 2020: GLOBOCAN estimates of incidence and mortality worldwide for 36 cancers in 185 countries. CA Cancer J Clin. (2021) 71:209–49. doi: 10.3322/caac.21660 33538338

[2] Kohler BA Sherman RL Howlader N Jemal A Ryerson AB Henry KA . Annual report to the nation on the status of cancer, 1975-2011, featuring incidence of breast cancer subtypes by race/ethnicity, poverty, and state. J Natl Cancer Inst. (2015) 107:djv048. doi: 10.1093/jnci/djv048 25825511 PMC4603551

[3] Sarhangi N Hajjari S Heydari SF Ganjizadeh M Rouhollah F Hasanzad M . Breast cancer in the era of precision medicine. Mol Biol Rep. (2022) 49:10023–37. doi: 10.1007/s11033-022-07571-2 35733061

[4] Waks AG Winer EP . Breast cancer treatment: A review. Jama. (2019) 321:288–300. doi: 10.1001/jama.2018.19323 30667505

[5] Zaha DC Lazăr E . Molecular characterization of apoptosis by the immunohistochemical evaluation of Bcl-2 in breast cancer. Rom J Morphol Embryol. (2012) 53:155–60. 22395515

[6] Chami M Prandini A Campanella M Pinton P Szabadkai G Reed JC . Bcl-2 and Bax exert opposing effects on Ca2+ signaling, which do not depend on their putative pore-forming region. J Biol Chem. (2004) 279:54581–9. doi: 10.1074/jbc.M409663200 15485871

[7] Yu B Sun X Shen HY Gao F Fan YM Sun ZJ . Expression of the apoptosis-related genes BCL-2 and BAD in human breast carcinoma and their associated relationship with chemosensitivity. J Exp Clin Cancer Res. (2010) 29:107. doi: 10.1186/1756-9966-29-107 20691103 PMC2924279

[8] Hassan WA El-Assmy M ElBanna AK Harbieh I Noufal N Lotfy H . Associations between BCL-2 expression and different histopathological prognostic factors in different molecular subtypes of invasive breast carcinoma of no special type. Histol Histopathol. (2024) 18831. doi: 10.14670/HH-18-831 39492626

[9] Zhu W Xu B . Overcoming resistance to endocrine therapy in hormone receptor-positive human epidermal growth factor receptor 2-negative (HR(+)/HER2(-)) advanced breast cancer: a meta-analysis and systemic review of randomized clinical trials. Front Med. (2021) 15:208–20. doi: 10.1007/s11684-020-0795-4 33175319

[10] Whittle JR Vaillant F Surgenor E Policheni AN Giner G Capaldo BD . Dual targeting of CDK4/6 and BCL2 pathways augments tumor response in estrogen receptor-positive breast cancer. Clin Cancer Res. (2020) 26:4120–34. doi: 10.1158/1078-0432.CCR-19-1872 32245900

[11] Nair BC Vadlamudi RK . Regulation of hormonal therapy resistance by cell cycle machinery. Gene Ther Mol Biol. (2008) 12:395. 20148177 PMC2817953

[12] Spring LM Wander SA Andre F Moy B Turner NC Bardia A . Cyclin-dependent kinase 4 and 6 inhibitors for hormone receptor-positive breast cancer: past, present, and future. Lancet. (2020) 395:817–27. doi: 10.1016/S0140-6736(20)30165-3 32145796

[13] Geum D Sun W Paik SK Lee CC Kim K . Estrogen-induced cyclin D1 and D3 gene expressions during mouse uterine cell proliferation *in vivo*: differential induction mechanism of cyclin D1 and D3. Mol Reprod Dev. (1997) 46:450–8. doi: 10.1002/(SICI)1098-2795(199704)46:4<450::AID-MRD2>3.0.CO;2-N 9094091

[14] Altucci L Addeo R Cicatiello L Germano D Pacilio C Battista T . Estrogen induces early and timed activation of cyclin-dependent kinases 4, 5, and 6 and increases cyclin messenger ribonucleic acid expression in rat uterus. Endocrinology. (1997) 138:978–84. doi: 10.1210/endo.138.3.5002 9048598

[15] Crozier L Foy R Adib R Kar A Holt JA Pareri AU . CDK4/6 inhibitor-mediated cell overgrowth triggers osmotic and replication stress to promote senescence. Mol Cell. (2023) 83:4062–77.e5. doi: 10.1016/j.molcel.2023.10.016 37977118

[16] Yang L Xue J Yang Z Wang M Yang P Dong Y . Side effects of CDK4/6 inhibitors in the treatment of HR+/HER2- advanced breast cancer: a systematic review and meta-analysis of randomized controlled trials. Ann Palliat Med. (2021) 10:5590–9. doi: 10.21037/apm-21-1096 34107710

[17] Johnston S Martin M Di Leo A Im SA Awada A Forrester T . MONARCH 3 final PFS: a randomized study of abemaciclib as initial therapy for advanced breast cancer. NPJ Breast cancer. (2019) 5:5. doi: 10.1038/s41523-018-0097-z 30675515 PMC6336880

[18] Sledge GW Jr. Toi M Neven P Neven P Sohn J Inoue K Pivot X . The effect of abemaciclib plus fulvestrant on overall survival in hormone receptor-positive, ERBB2-negative breast cancer that progressed on endocrine therapy-MONARCH 2: A randomized clinical trial. JAMA Oncol. (2020) 6:116–24. doi: 10.1001/jamaoncol.2019.4782 PMC6777264 31563959

[19] Zhang QY Sun T Yin YM Li HP Yan M Tong ZS . MONARCH plus: abemaciclib plus endocrine therapy in women with HR+/HER2- advanced breast cancer: the multinational randomized phase III study. Ther Adv Med Oncol. (2020) 12:1758835920963925. doi: 10.1177/1758835920963925 33149768 PMC7586037

[20] Xu B Zhang Q Zhang P Hu X Li W Tong Z . Dalpiciclib or placebo plus fulvestrant in hormone receptor-positive and HER2-negative advanced breast cancer: a randomized, phase 3 trial. Nat Med. (2021) 27:1904–9. doi: 10.1038/s41591-021-01562-9 34737452

[21] Zhang P Zhang Q Tong Z Sun T Li W Ouyang Q . Dalpiciclib plus letrozole or anastrozole versus placebo plus letrozole or anastrozole as first-line treatment in patients with hormone receptor-positive, HER2-negative advanced breast cancer (DAWNA-2): a multicentre, randomised, double-blind, placebo-controlled, phase 3 trial. Lancet Oncol. (2023) 24:646–57. doi: 10.1016/S1470-2045(23)00172-9 37182538

[22] Hortobagyi GN Stemmer SM Burris HA Yap YS Sonke GS Hart L . Overall survival with ribociclib plus letrozole in advanced breast cancer. New Engl J Med. (2022) 386:942–50. doi: 10.1056/NEJMoa2114663 35263519

[23] Lu YS Im SA Colleoni M Franke F Bardia A Cardoso F . Updated Overall Survival of Ribociclib plus Endocrine Therapy versus Endocrine Therapy Alone in Pre- and Perimenopausal Patients with HR+/HER2- Advanced Breast Cancer in MONALEESA-7: a Phase III Randomized Clinical Trial. Clin Cancer Res. (2022) 28:851–9. doi: 10.1158/1078-0432.CCR-21-3032 PMC9377723 34965945

[24] Neven P Fasching PA Chia S Jerusalem G De Laurentiis M Im SA . Updated overall survival from the MONALEESA-3 trial in postmenopausal women with HR+/HER2- advanced breast cancer receiving first-line ribociclib plus fulvestrant. Breast Cancer Res. (2023) 25:103. doi: 10.1186/s13058-023-01701-9 37653397 PMC10469877

[25] Albanell J Martínez MT Ramos M O'Connor M de la Cruz-Merino L Santaballa A . Randomized phase II study of fulvestrant plus palbociclib or placebo in endocrine-sensitive, hormone receptor-positive/HER2-advanced breast cancer: GEICAM/2014-12 (FLIPPER). Eur J Cancer (Oxford England: 1990). (2022) 161:26–37. doi: 10.1016/j.ejca.2021.11.010 34902765

[26] Finn RS Martin M Rugo HS Jones S Im SA Gelmon K . Palbociclib and letrozole in advanced breast cancer. New Engl J Med. (2016) 375:1925–36. doi: 10.1056/NEJMoa1607303 27959613

[27] Finn RS Boer K Bondarenko I Patel R Pinter T Schmidt M . Overall survival results from the randomized phase 2 study of palbociclib in combination with letrozole versus letrozole alone for first-line treatment of ER+/HER2- advanced breast cancer (PALOMA-1, TRIO-18). Breast Cancer Res Treat. (2020) 183:419–28. doi: 10.1007/s10549-020-05755-7 PMC7383036 32683565

[28] Turner NC Slamon DJ Ro J Bondarenko I Im SA Masuda N . Overall survival with palbociclib and fulvestrant in advanced breast cancer. New Engl J Med. (2018) 379:1926–36. doi: 10.1056/NEJMoa1810527 30345905

[29] Xu B Hu X Li W Sun T Shen K Wang S . Palbociclib plus letrozole versus placebo plus letrozole in Asian postmenopausal women with oestrogen receptor-positive/human epidermal growth factor receptor 2-negative advanced breast cancer: primary results from PALOMA-4. Eur J Cancer (Oxford England: 1990). (2022) 175:236–45. doi: 10.1016/j.ejca.2022.08.012 36155117

[30] O’Leary B Finn RS Turner NC . Treating cancer with selective CDK4/6 inhibitors. Nat Rev Clin Oncol. (2016) 13:417–30. doi: 10.1038/nrclinonc.2016.26 27030077

[31] Tate SC Cai S Ajamie RT Burke T Beckmann RP Chan EM . Semi-mechanistic pharmacokinetic/pharmacodynamic modeling of the antitumor activity of LY2835219, a new cyclin-dependent kinase 4/6 inhibitor, in mice bearing human tumor xenografts. Clin Cancer Res. (2014) 20:3763–74. doi: 10.1158/1078-0432.CCR-13-2846 24850847

[32] Gelbert LM Cai S Lin X Sanchez-Martinez C Del Prado M Lallena MJ . Preclinical characterization of the CDK4/6 inhibitor LY2835219: *in-vivo* cell cycle-dependent/independent anti-tumor activities alone/in combination with gemcitabine. Invest New Drugs. (2014) 32:825–37. doi: 10.1007/s10637-014-0120-7 PMC4169866 24919854

[33] Lee RJ Albanese C Fu M D'Amico M Lin B Watanabe G . Cyclin D1 is required for transformation by activated Neu and is induced through an E2F-dependent signaling pathway. Mol Cell Biol. (2000) 20:672–83. doi: 10.1128/MCB.20.2.672-683.2000 PMC85165 10611246

[34] Thangavel C Dean JL Ertel A Knudsen KE Aldaz CM Witkiewicz AK . Therapeutically activating RB: reestablishing cell cycle control in endocrine therapy-resistant breast cancer. Endocr Relat Cancer. (2011) 18:333–45. doi: 10.1530/ERC-10-0262 PMC3624623 21367843

[35] Rader J Russell MR Hart LS Nakazawa MS Belcastro LT Martinez D . Dual CDK4/CDK6 inhibition induces cell-cycle arrest and senescence in neuroblastoma. Clin Cancer Res. (2013) 19:6173–82. doi: 10.1158/1078-0432.CCR-13-1675 PMC3844928 24045179

[36] Zhang YX Sicinska E Czaplinski JT Remillard SP Moss S Wang Y . Antiproliferative effects of CDK4/6 inhibition in CDK4-amplified human liposarcoma *in vitro* and *in vivo* . Mol Cancer Ther. (2014) 13:2184–93. doi: 10.1158/1535-7163.MCT-14-0387 25028469

[37] Dai Q Wang Y Liao M Chen H . Efficacy and safety of CDK4/6 inhibitors combined with endocrine therapy versus endocrine therapy alone in hormone receptor-positive, HER2-negative, advanced breast cancer: a systematic review and meta-analysis. Ann Palliat Med. (2022) 11:3727–42. doi: 10.21037/apm-22-1306 36635998

[38] Brandão M Maurer C Ziegelmann PK Pondé NF Ferreira A Martel S . Endocrine therapy-based treatments in hormone receptor-positive/HER2-negative advanced breast cancer: systematic review and network meta-analysis. ESMO Open. (2020) 5. doi: 10.1136/esmoopen-2020-000842 PMC7451473 32847835

[39] Park YH Kim TY Kim GM Kang SY Park IH Kim JH . Palbociclib plus exemestane with gonadotropin-releasing hormone agonist versus capecitabine in premenopausal women with hormone receptor-positive, HER2-negative metastatic breast cancer (KCSG-BR15-10): a multicentre, open-label, randomised, phase 2 trial. Lancet Oncol. (2019) 20:1750–9. doi: 10.1016/S1470-2045(19)30565-0 31668850

[40] Prat A Saura C Pascual T Hernando C Muñoz M Paré L . Ribociclib plus letrozole versus chemotherapy for postmenopausal women with hormone receptor-positive, HER2-negative, luminal B breast cancer (CORALLEEN): an open-label, multicentre, randomised, phase 2 trial. Lancet Oncol. (2020) 21:33–43. doi: 10.1016/S1470-2045(19)30786-7 31838010

[41] Wang X Zhao S Xin Q Zhang Y Wang K Li M . Recent progress of CDK4/6 inhibitors’ current practice in breast cancer. Cancer Gene Ther. (2024) 31:1283–91. doi: 10.1038/s41417-024-00747-x PMC11405274 38409585

[42] Tan AR Wright GS Thummala AR Danso MA Popovic L Pluard TJ . Trilaciclib plus chemotherapy versus chemotherapy alone in patients with metastatic triple-negative breast cancer: a multicentre, randomised, open-label, phase 2 trial. Lancet Oncol. (2019) 20:1587–601. doi: 10.1016/S1470-2045(19)30616-3 31575503

[43] Mita MM Joy AA Mita A Sankhala K Jou YM Zhang D . Randomized phase II trial of the cyclin-dependent kinase inhibitor Dinaciclib (MK-7965) versus capecitabine in patients with advanced breast cancer. Clin Breast Cancer. (2014) 14:169–76. doi: 10.1016/j.clbc.2013.10.016 24393852

[44] Ramos-Esquivel A Hernández-Steller H Savard MF Savard MF Landaverde DU . Cyclin-dependent kinase 4/6 inhibitors as first-line treatment for post-menopausal metastatic hormone receptor-positive breast cancer patients: a systematic review and meta-analysis of phase III randomized clinical trials. Breast Cancer. (2018) 25:479–88. doi: 10.1007/s12282-018-0848-6 29470723

[45] Rugo HS Huober J García-Sáenz JA Masuda N Sohn JH Andre VAM . Management of abemaciclib-associated adverse events in patients with hormone receptor-positive, human epidermal growth factor receptor 2-negative advanced breast cancer: safety analysis of MONARCH 2 and MONARCH 3. Oncologist. (2021) 26:e53–65. doi: 10.1002/onco.13531 PMC7794176 32955138

[46] Desnoyers A Nadler MB Kumar V Kumar V Saleh R Amir E . Comparison of treatment-related adverse events of different Cyclin-dependent kinase 4/6 inhibitors in metastatic breast cancer: A network meta-analysis. Cancer Treat Rev. (2020) 90:102086. doi: 10.1016/j.ctrv.2020.102086 32861975

[47] Smith TJ Bohlke K Lyman GH Carson KR Crawford J Cross SJ . Recommendations for the use of WBC growth factors: american society of clinical oncology clinical practice guideline update. J Clin Oncol. (2015) 33:3199–212. doi: 10.1200/JCO.2015.62.3488 26169616

[48] Shu Y Wang L Ding Y Zhang Q . Disproportionality analysis of abemaciclib in the FDA adverse event reporting system: A real-world post-marketing pharmacovigilance assessment. Drug Saf. (2023) 46:881–95. doi: 10.1007/s40264-023-01334-z 37418089

[49] Stark AM Pfannenschmidt S Tscheslog H Maass N Rösel F Mehdorn HM . Reduced mRNA and protein expression of BCL-2 versus decreased mRNA and increased protein expression of BAX in breast cancer brain metastases: a real-time PCR and immunohistochemical evaluation. Neurol Res. (2006) 28:787–93. doi: 10.1179/016164106X110364 17288732

[50] Turner NC Liu Y Zhu Z Loi S Colleoni M Loibl S . Cyclin E1 expression and palbociclib efficacy in previously treated hormone receptor-positive metastatic breast cancer. J Clin Oncol. (2019) 37:1169–78. doi: 10.1200/JCO.18.00925 PMC6506420 30807234

