# Supplementary material for: A meta-analysis and systematic review of different cyclin-dependent kinase 4/6 inhibitors in breast cancer
Source: Front Oncol. 2025 Mar 4;15:1472407. doi: 10.3389/fonc.2025.1472407 (PMC11913826; doi:10.3389/fonc.2025.1472407)
Supplement: Supplementary file 1 [file Table1.docx]

**Search strategy by November 30, 2023**

**PICOS**

**P:**

**MeSH terms:** **Breast Neoplasms**

**Free terms:** (Breast Neoplasm) OR (Breast Tumor) OR (Breast Cancer) OR (Breast Carcinoma) OR (Mammary Cancer) OR (Mammary Carcinoma) OR (Mammary Neoplasm) OR (Mammary Tumor)

**I:** (Cyclin-dependent kinase 4/6) OR (CDK4/6) OR (Abemaciclib) OR (Palbociclib) OR (Dalpiciclib) OR (Trilaciclib) OR (Ribociclib)

**C:**

**O:**

**S:**

| **Database** | **Retrieval strategy** | **Number** |
| --- | --- | --- |
| **Pubmed** | #1 " Breast Neoplasms "[Mesh] 346898  #2 (Breast Neoplasm [Title/Abstract])  #3 (Breast Tumor [Title/Abstract])  #4 (Breast Cancer [Title/Abstract])  #5 (Breast Carcinoma [Title/Abstract])  #6 (Mammary Cancer [Title/Abstract])  #7 (Mammary Carcinoma [Title/Abstract])  #8 (Mammary Neoplasm [Title/Abstract])  #9 (Mammary Tumor [Title/Abstract])  #10 #1 OR #2 OR #3 OR #4 OR #5 OR #6 OR #7 OR #8 OR #9 539777  #11 (Cyclin-dependent kinase 4/6 [Title/Abstract])  #12 (CDK4/6 [Title/Abstract])  #13 (Abemaciclib [Title/Abstract])  #14 (Palbociclib [Title/Abstract])  #15 (Dalpiciclib [Title/Abstract])  #16 (Trilaciclib [Title/Abstract])  #17 (Ribociclib [Title/Abstract])  #18 #11 OR #12 OR #13 OR #14 OR #15 OR #16 OR #17 4617  #19 #10 AND #18 2622 | 2622 |
| **Cochrane Library** | #1 MeSH descriptor: [Breast Neoplasms] explode all trees 18209  #2 (Breast Neoplasm):ti,ab,kw  #3 (Breast Tumor):ti,ab,kw  #4 (Breast Cancer):ti,ab,kw  #5 (Breast Carcinoma):ti,ab,kw  #6 (Mammary Cancer):ti,ab,kw  #7 (Mammary Carcinoma):ti,ab,kw  #8 (Mammary Neoplasm):ti,ab,kw  #9 (Mammary Tumor):ti,ab,kw  #10 #1 OR #2 OR #3 OR #4 OR #5 OR #6 OR #7 OR #8 OR #9 46774  #11 (Cyclin-dependent kinase 4/6 ):ti,ab,kw  #12 (CDK4/6):ti,ab,kw  #13 (Abemaciclib):ti,ab,kw  #14 (Palbociclib):ti,ab,kw  #15 (Dalpiciclib ):ti,ab,kw  #16 (Trilaciclib ):ti,ab,kw  #17 (Ribociclib ):ti,ab,kw  #18 #11 OR #12 OR #13 OR #14 OR #15 OR #16 OR #17 1399  #19 #10 AND #18 1206 | 1206 |
| **Embase** | #1 ' Breast Neoplasms '/exp 688123  #2 (Breast Neoplasm):ab,ti  #3 (Breast Tumor):ab,ti  #4 (Breast Cancer):ab,ti  #5 (Breast Carcinoma):ab,ti  #6 (Mammary Cancer):ab,ti  #7 (Mammary Carcinoma):ab,ti  #8 (Mammary Neoplasm):ab,ti  #9 (Mammary Tumor):ab,ti  #10 #1 OR #2 OR #3 OR #4 OR #5 OR #6 OR #7 OR #8 OR #9 852505  #11 (Cyclin-dependent kinase 4/6 ):ab,ti  #12 (CDK4/6):ab,ti  #13 (Abemaciclib):ab,ti  #14 (Palbociclib):ab,ti  #15 (Dalpiciclib ):ab,ti  #16 (Trilaciclib ):ab,ti  #17 (Ribociclib ):ab,ti  #18 #11 OR #12 OR #13 OR #14 OR #15 OR #16 OR #17 79904  #19 #10 AND #18 14101 | 14101 |
